# Global transcriptional responses to the bacteriocin colicin M in *Escherichia coli*

**DOI:** 10.1186/1471-2180-13-42

**Published:** 2013-02-19

**Authors:** Simona Kamenšek, Darja Žgur-Bertok

**Affiliations:** 1 Department of Biology, Biotechnical Faculty, University of Ljubljana, 1000, Ljubljana, Slovenia

**Keywords:** Antimicrobial agent, Bacteriocin, Colicin M, *Escherichia coli*, Gene expression, Peptidoglycan

## Abstract

**Background:**

Bacteriocins are protein antimicrobial agents that are produced by all prokaryotic lineages. *Escherichia coli* strains frequently produce the bacteriocins known as colicins. One of the most prevalent colicins, colicin M, can kill susceptible cells by hydrolyzing the peptidoglycan lipid II intermediate, which arrests peptidoglycan polymerization steps and provokes cell lysis. Due to the alarming rise in antibiotic resistance and the lack of novel antimicrobial agents, colicin M has recently received renewed attention as a promising antimicrobial candidate. Here the effects of subinhibitory concentrations of colicin M on whole genome transcription in *E. coli* were investigated, to gain insight into its ecological role and for purposes related to antimicrobial therapy.

**Results:**

Transcriptome analysis revealed that exposure to subinhibitory concentrations of colicin M altered expression of genes involved in envelope, osmotic and other stresses, including genes of the CreBC two-component system, exopolysaccharide production and cell motility. Nonetheless, there was no induction of biofilm formation or genes involved in mutagenesis.

**Conclusion:**

At subinhibitory concentrations colicin M induces an adaptive response primarily to protect the bacterial cells against envelope stress provoked by peptidoglycan damage. Among the first induced were genes of the CreBC two-component system known to promote increased resistance against colicins M and E2, providing novel insight into the ecology of colicin M production in natural environments. While an adaptive response was induced nevertheless, colicin M application did not increase biofilm formation, nor induce SOS genes, adverse effects that can be provoked by a number of traditional antibiotics, providing support for colicin M as a promising antimicrobial agent.

## Background

In their natural environments, bacteria are frequently exposed to various stresses, including antimicrobials. It has been generally assumed that the role of antibiotics in nonclinical environments is the inhibition of competitors. Nevertheless, antibiotic concentrations in natural habitats can be variable, with high concentrations only in the vicinity of the producer. Recent studies have shown that antibiotics can act in a concentration-dependent manner that exhibits dual ecological roles: (i) at high concentrations they can destroy microorganisms; while (ii) at low concentrations they can modulate bacterial gene expression to promote ecological adaptation [1,2].

Bacteriocins are ribosomally synthesized antimicrobial agents that are produced by all prokaryotic lineages and they are generally active against closely related species. Among the best characterized bacteriocins are those produced by *Escherichia coli*, which are known as colicins. The majority of colicins act by membrane permeabilization, followed by nuclease activity, while one colicin, colicin M, inhibits peptidoglycan synthesis.

Uptake of colicin M proceeds by binding to the FhuA outer membrane receptor followed by energy-dependent translocation into the periplasm through the TonB system (TonB, ExbB and ExbD) and the proton motive force of the inner membrane [3]. Colicin M is a phosphotase that cleaves the undecaprenyl-phosphate-linked peptidoglycan precursor, lipid II producing free undecaprenol and 1-pyrophospho-Mur-GlCNAc-pentapeptide. In the periplasm, hydrolysis of peptidoglycan lipid precursors results in arrest of polymerization steps and cell lysis [4]. Operons that encode colicin M and B are tightly linked on large conjugative plasmids [5,6], and these are among the most abundant colicins produced by *E. coli* strains [7].

A number of studies have been aimed at defining the function of colicins in microbial communities. They might serve to enable invasion or defense of an ecological niche [8]. They have been shown to mediate population and community level interactions, promoting microbial diversity within *E. coli* populations in the mammalian colon [9]. To obtain more insight into the ecological roles of one of the most prevalent colicins, the effects of subinhibitory concentrations of colicin M on genome wide transcription in *E. coli* was studied.

Antibiotic resistance currently represents one of the greatest worldwide threats to human health therefore, novel antibiotics are urgently needed. Antibiotic resistance among the Enterobacteriaceae represents a particular threat [10,11]. As colicin M promotes the irreversible hydrolysis of lipid II, a peptidoglycan lipid intermediate that is common to all bacteria, it is also a promising candidate for development of a novel antimicrobial agent [12]. Analysis of the gene expression profile was thus also undertaken, to acquire insight into adaptive responses to colicin M that might be detrimental during antimicrobial therapy.

## Results and discussion

### Transcriptome analysis of *E. coli* MG1655 exposed to subinhibitory concentrations of colicin M

**Figure 1:**
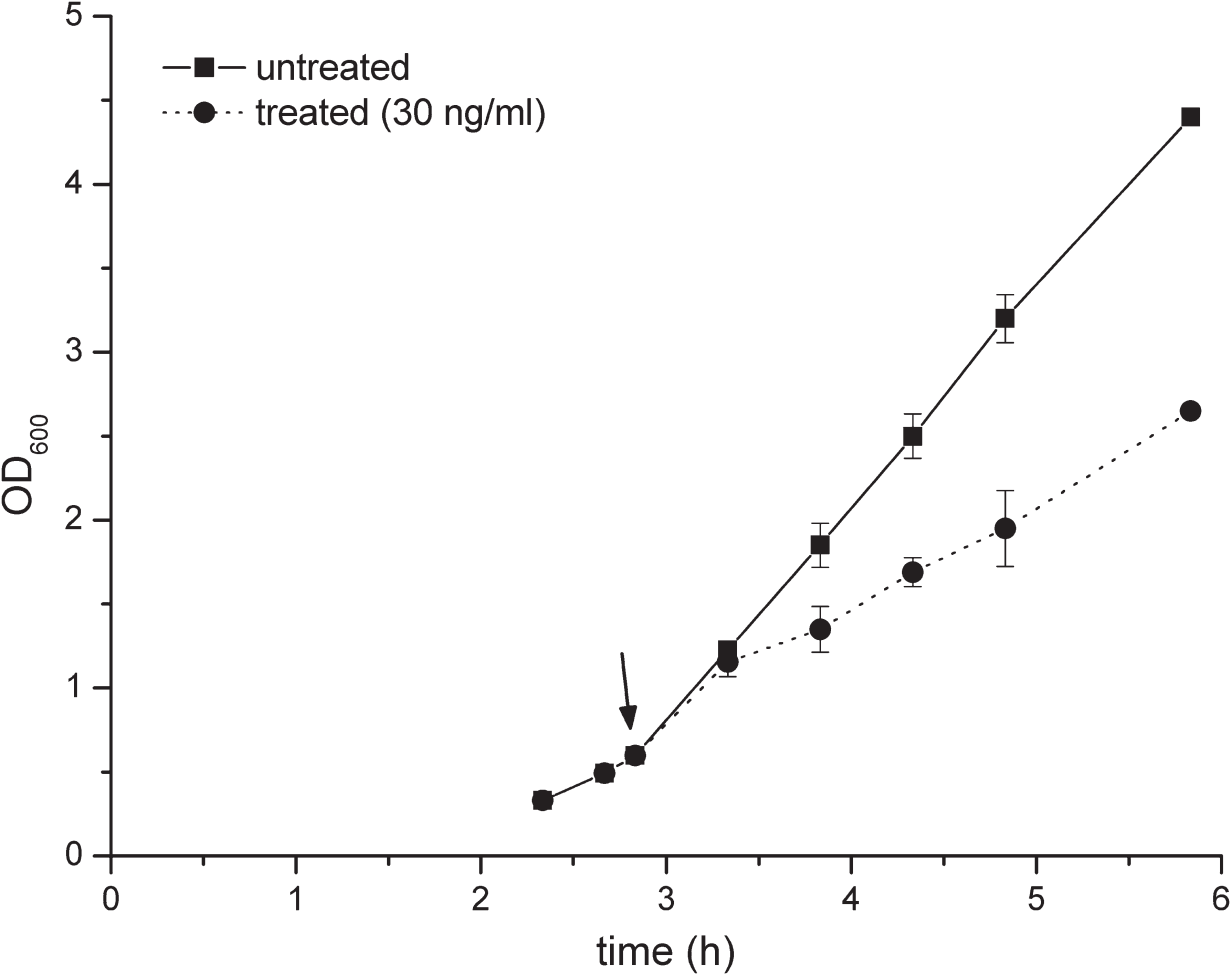
**Growth of MG1655 without and with colicin M.** The arrow denotes the time of addition of colicin M at subinhibitory concentrations (30 ng/ml). The experiment was performed three times, and the means ± standard errors of the means (error bars) are shown.

The effects of colicin M on whole genome transcription of *E. coli* MG1655, a laboratory strain with minimal genetic manipulation that approximates the wild type [13], was investigated by microarray analysis. To choose the appropriate conditions for determing the colicin M induced transcriptome, mid-exponential phase cultures of strain MG1665 were exposed to various concentrations of colicin M and the growth response was monitored. On the basis of these results a concentration of 30 ng/ml was determined as subinhibitory and chosen for transcriptome analysis. To focus on stress responses provoked by colicin M, samples were collected as growth slowed, 30 min following exposure to colicin M and upon regrowth, 60 min following exposure to colicin M, Figure 1 (see also Additional file 1: Figure S1 and Additional file 2: Figure S2).

**Figure 2:**
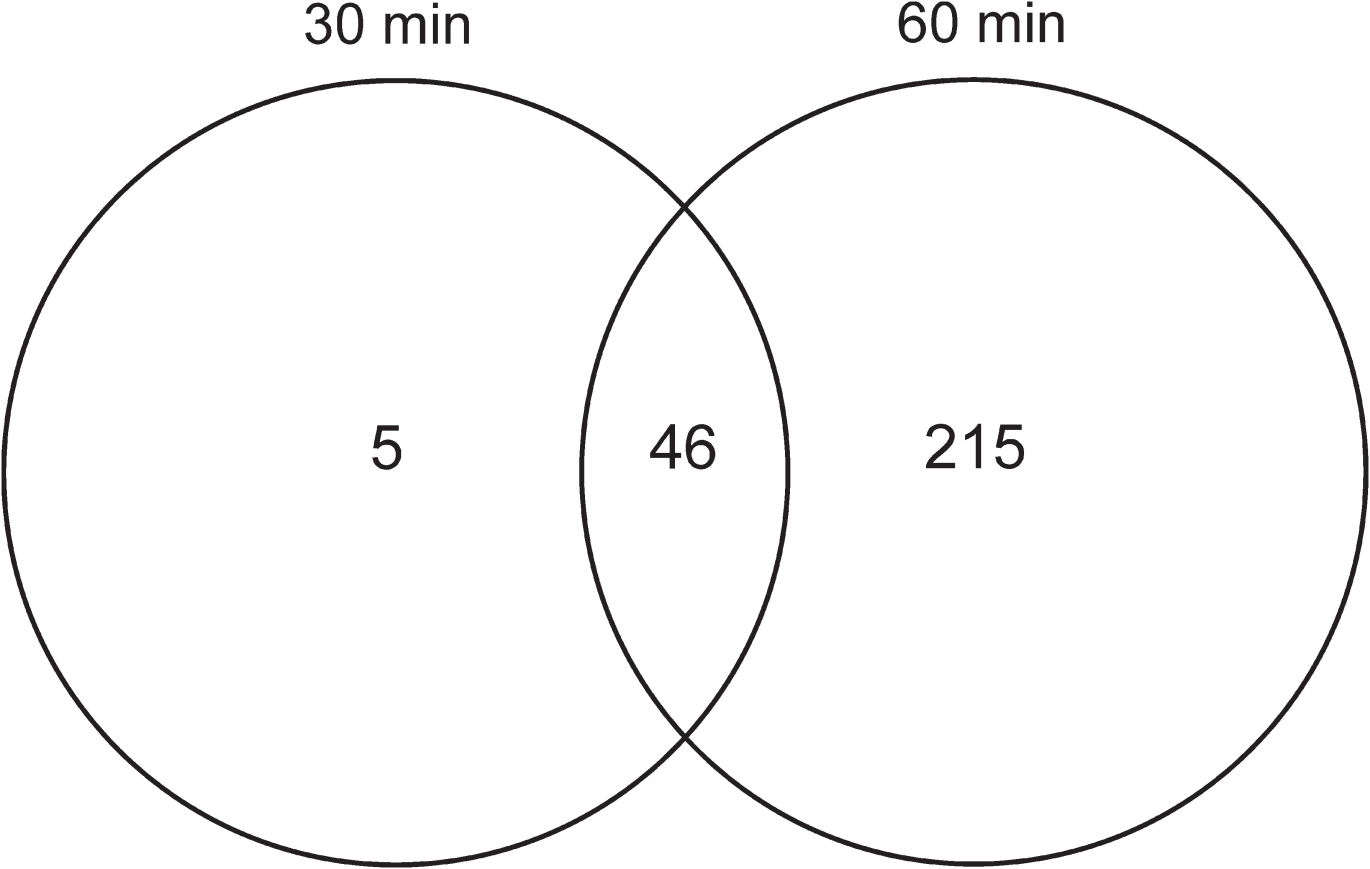
**Venn diagram of gene expression in 30 min and 60 min treated *E. coli *MG1655.** Time course analysis of differentially expressed genes, reveals number of genes induced following 30 min and 60 min exposure to subinhibitory concentrations of colicin M.

The 30 min exposure up-regulated the expression of 49 genes, with 2 genes down-regulated (log_2_ fold change >1 and < −1, *P* ≤0.05). On the other hand, the 60-min exposure to colicin M significantly up-regulated the expression of 210 genes, with expression of 51 genes down-regulated (log_2_ fold change >1 and < −1, *P* ≤0.05). Time course analysis showed that 46 genes were differentially expressed following 30 and 60 min colicin M treatment while 5 were differentially expressed only after 30 min treatment, (Figure 2). Whereas 30 min exposure provoked differential expression of a limited number of genes across several gene groups, more genes were altered in their expression (extensive transcriptional changes were observed) following 60 min treatment. Among the first significantly induced genes were those of two component sensory systems and several genes encoding membrane proteins.

Time course analysis of differential gene expression, after 30 and 60 min treatment, is presented in Additional file 3: Table S1 (log_2_ fold change >1 and < −1, *P* ≤0.05). Genes considered for interpretation are presented in Table 1 and are described below.

**Table 1:** Genes with modulated expression after exposure to colicin M over time, 30 and 60 min

**Category/Gene symbol**	**Gene accession No.**	**Gene description**	**30 min log**_**2 **_**ratio**	**60 min log**_**2 **_**ratio**
**Envelope stress regulators/systems**				
*rcsA*	946467	DNA-binding transcriptional activator, co-regulator with RcsB	**3.38**	**6.13**
*cpxP*	2847688	inhibitor of the cpx response; periplasmic adaptor protein	**1.57**	**2.61**
*pspA*	945887	regulatory protein for phage-shock-protein operon	**1.35**	**1.18**
*pspB*	945893	DNA-binding transcriptional regulator of psp operon	**1.32**	**1.47**
*pspC*	945499	DNA-binding transcriptional activator	**1.14**	**1.52**
*pspD*	945635	peripheral inner membrane phage-shock protein	*0.83*	**1.78**
*pspG*	948557	phage shock protein G	**1.55**	**2.29**
**Colanic acid biosynthetic process**				
*wza*	946558	lipoprotein required for capsular polysaccharide translocation through the outer membrane	**3.59**	**7.12**
*wzb*	946564	protein-tyrosine phosphatase	**2.44**	**6.33**
*wzc*	946567	protein-tyrosine kinase	**1.52**	**6.72**
*wcaA*	946570	predicted glycosyl transferase	*0.93*	**5.7**
*wcaB*	946573	predicted acyl transferase	*0.69*	**5.73**
*wcaC*	946579	predicted glycosyl transferase	*0.56*	**5.47**
*wcaD*	946550	predicted colanic acid polymerase	*0.78*	**7.23**
*wcaE*	946543	predicted glycosyl transferase	**1.25**	**7.26**
*wcaF*	946578	predicted acyl transferase	*0.97*	**7.21**
*gmd*	946562	GDP-D-mannose dehydratase, NAD(P)-binding	0.71	**6.65**
*fcl*	946563	bifunctional GDP-fucose synthetase: GDP-4-dehydro-6-deoxy-D-mannose epimerase/GDP-4-dehydro-6-L-deoxygalactose reductase	*0.32*	**6.57**
*gmm*	946559	GDP-mannose mannosyl hydrolase	*0.3*	**6.15**
*wcaI*	946588	predicted glycosyl transferase	*0.3*	**5.92**
*cpsG*	946574	phosphomannomutase	*0.09*	**5.15**
*cpsB*	946580	mannose-1-phosphate guanyltransferase	*0.26*	**5.1**
*wcaJ*	946583	predicted UDP-glucose lipid carrier transferase	*0.11*	**4.82**
*wzxC*	946581	predicted colanic acid exporter	*0.1*	**4.45**
*wcaK*	946569	Colanic acid biosynthesis protein	*−0.12*	**4.45**
*wcaL*	946565	predicted glycosyl transferase	*−0.13*	**3.63**
*manA*	944840	mannose-6-phosphate isomerase	*0.19*	**1.05**
*ugd*	946571	UDP-glucose 6-dehydrogenase	*0.46*	**4.36**
*wcaM*	946561	colanic acid biosynthesis protein	*−0.01*	**2.71**
*galU*	945730	glucose-1-phosphate uridylyltransferase	*0.44*	**1.4**
**Extracellular polysaccharide distinct from colanic acid**				
*yjbE*	948534	predicted protein	**1.55**	**5.74**
*yjbF*	948533	predicted lipoprotein	**1.73**	**5.67**
*yjbG*	948526	conserved protein	*0.67*	**4.29**
*yjbH*	948527	predicted porin	*0.66*	**5.23**
**Peptidoglycan synthesis**				
*anmK*	946810	anhydro-N-acetylmuramic acid kinase	*0.16*	**1.17**
*mrcB*	944843	fused glycosyl transferase and transpeptidase	*0.47*	**1.01**
*ycfS*	945666	L,D-transpeptidase linking Lpp to murein	0.77	**2**
**Osmotic stress response**				
*osmB*	945866	lipoprotein	**2.41**	**2.95**
*osmC*	946043	osmotically inducible, stress-inducible membrane protein	*0.44*	**1.15**
*opgB*	948888	phosphoglycerol transferases I and II	*0.12*	**1.27**
*opgC*	946944	membrane protein required for succinylation of osmoregulated periplasmic glucans (OPGs)	*0.31*	**1.85**
*ivy*	946530	inhibitor of vertebrate C-lysozyme	**1.55**	**1.26**
*mliC*	946811	inhibitor of C-lysozyme, membrane-bound; predicted lipoprotein	**2.17**	**3.92**
*ybdG*	946243	predicted mechanosensitive channel	*0.69*	**1.26**
*dppB*	948063	dipeptide/heme transporter	*−0.29*	**3.29**
*dppF*	948056	dipeptide transporter	*−0.1*	**2.33**
*dppC*	948064	dipeptide/heme transporter	*−0.09*	**2.33**
*dppD*	948065	dipeptide/heme transporter	*−0.09*	**2.1**
*dppA*	948062	dipeptide transporter	*0.02*	**1.13**
**Other stress responses**				
*ydeI*	946068	conserved protein	**1.99**	**3.96**
*treR*	948760	DNA-binding transcriptional repressor	*0.65*	**1.88**
*ibpA*	948200	heat shock chaperone	*−0.01*	**1.78**
*ibpB*	948192	heat shock chaperone	*0.02*	**2.9**
*hslJ*	946525	heat-inducible lipoprotein involved in novobiocin resistance	**2.33**	**3.32**
*yhbO*	947666	predicted intracellular protease	**2.29**	**2.67**
*iraM*	945729	RpoS stabilizer during Mg starvation, anti-RssB factor	*0.33*	**1.6**
*creD*	948868	inner membrane protein	**5.66**	**4.96**
*cbrB*	948231	inner membrane protein, creBC regulon	**5.2**	**4.29**
*cbrA*	948197	predicted oxidoreductase with FAD/NAD(P)-binding domain	**4.3**	**3.35**
*cbrC*	948230	conserved protein, UPF0167 family	**3.77**	**2.8**
*spy*	946253	envelope stress induced periplasmic protein	**1.71**	**2.99**
*htpX*	946076	predicted endopeptidase	*0.27*	**1.01**
*yggG*	945173	heat shock protein binding to Era protein; predicted peptidase	**1.01**	**1.82**
**Biofilm formation**				
*ycfJ*	945977	predicted protein	**4.77**	**5.8**
*rprA*	2847671	ncRNA	**3.86**	**4.85**
*omrA*	2847746	ncRNA	*0.36*	**1.76**
*omrB*	2847747	ncRNA	*0.77*	**1.74**
*bdm*	946041	biofilm-dependent modulation protein	**4.49**	**4.21**
*ydeH*	946075	diguanylate cyclase, required for pgaD induction	**1.38**	**1.68**
**Cell motility**				
*fliZ*	946833	RpoS antagonist; putative regulator of FliA activity	*−0.41*	**−1.05**
*fliE*	946446	flagellar basal-body component	*−0.66*	**−1.07**
*fliG*	946451	flagellar motor switching and energizing component	*−0.28*	**−1.07**
*flgN*	945634	export chaperone for FlgK and FlgL	*−0.29*	**−1.12**
*flgA*	946300	assembly protein for flagellar basal-body periplasmic P ring	*−0.09*	**−1.17**
*flgF*	945639	flagellar component of cell-proximal portion of basal-body rod	*−0.29*	**−1.21**
*flgM*	946684	anti-sigma factor for FliA (sigma 28)	*−0.27*	**−1.23**
*fliA*	948824	RNA polymerase, sigma 28 (sigma F) factor	*−0.21*	**−1.45**
*flgD*	945813	flagellar hook assembly protein	*−0.33*	**−1.61**
*flgE*	945636	flagellar hook protein	*−0.05*	**−1.72**
*flgC*	946687	flagellar component of cell-proximal portion of basal-body rod	*−0.04*	**−2.14**
*flgB*	945678	flagellar component of cell-proximal portion of basal-body rod	*−0.19*	**−2.4**
*flhC*	947280	DNA-binding transcriptional dual regulator with FlhD	*−0.76*	**−2.54**
*flhD*	945442	DNA-binding transcriptional dual regulator with FlhC	*−0.76*	**−2.54**
**Amino acid transport/acid resistance**				
*glnP*	945621	glutamine transporter subunit	*−0.23*	**−1.17**
*gadB*	946058	glutamate decarboxylase B, PLP-dependent	*0.03*	**−1.18**
*glnQ*	945435	glutamine transporter subunit	*−0.15*	**−1.25**
*glnG*	948361	fused DNA-binding response regulator in two-component regulatory system with GlnL: response regulator/sigma54 interaction protein	*−0.15*	**−1.32**
*gadA*	948027	glutamate decarboxylase A, PLP-dependent	*−0.23*	**−1.64**
*gadE*	948023	DNA-binding transcriptional activator	*0.13*	**−1.38**
*slp*	948022	outer membrane lipoprotein	*−0.18*	**−1.91**
*hdeB*	948026	acid-resistance protein	*0.13*	**−1.17**
*hdeD*	948024	acid-resistance membrane protein	*−0.01*	**−1.04**
**Poorly characterized**				
*ymgD*	945732	predicted protein	**3.45**	**3.65**
*ymgG*	945728	conserved protein, UPF0757 family	**3.87**	**3.55**
*yfdC*	944801	predicted inner membrane protein	**1.02**	**2.25**
*yjbJ*	948553	conserved protein, UPF0337 family	**0.97**	**1.19**
*yaaX*	944747	predicted protein	***1.59***	**4.12**
*yegS*	946626	phosphatidylglycerol kinase, metal-dependent	0.81	**1.65**
*yaiY*	945223	inner membrane protein, DUF2755 family	**3.94**	**5.22**

(bold indicates genes with gene expression log_2_ ratio (fold change) ≥1 and ≤−1, denoting fold change ≥2 or ≤−2, and with p≤0.05, and italic indicates genes with p≥0.05).

### Colicin M treatment affects signal transduction pathways

The bacterial envelope protects the bacterial cell from external stress and performs essential functions such as, transport of nutrients and waste, as well as respiration and adhesion. In Gram-negative bacteria the outer membrane acts as a permeability barrier, with porins for transport and protection against phagocytosis [14] while processes central for energy generation and nutrient transport occur in the inner membrane [15]. In the periplasmic space peptidoglycan ensures the structural integrity of the cell by preventing osmolysis. To sense and rapidly respond to environmental signals, bacteria primarily use two-component signal transduction systems, composed of an inner membrane histidine kinase and a cytoplasmic response regulator [16].

Peptidoglycan is the major structural component of the bacterial cell wall. It provides the bacterial cell structural strength and protects the osmotically sensitive membrane [17]. As expected, by targeting peptidoglycan synthesis, colicin M induced an envelope stress response. In *E. coli* envelope homeostasis is monitored by several stress responses; namely, Rcs, Cpx, Psp, σ^E^, Bae and vesicle release responses [18-20]. Our microarray analysis revealed that the Rcs regulon has a major role in the response to envelope stress induced by colicin M. RcsC and RcsD are inner-membrane-bound proteins, with RcsC as the sensor kinase that autophosphorylates upon sensing the appropriate signal, while RcsD transfers the phosphoryl group to the transcriptional regulator RcsB. Certain promoters require the RcsA protein to act as an auxiliary regulatory protein, apparently exerting its effects by forming a heterodimer with RcsB [21]. The Rcs system controls the production of exopolysaccharides [22], biofilm formation [23,24], cell motility, and chemotaxis [25]. We observed induction of *rcsA* and a number of other Rcs-regulated genes: the exopolysaccharide operons *wca* for colanic acid synthesis, and *yjbEFGH*, as well as genes *osmB*, *ymgG* and *ymgD*, *ivy*, *yfbR*, *ugd*, *yfdC*, *yjbJ*, *galU*, *yaaX*, *yggG*, *yegS*, *spy*, *rprA, bdm* and *yaiY* (Table 1)*.* Recently, perturbations to peptidoglycan by several ß-lactam antibiotics were shown to elicit shared as well as unique responses with all activating the Rcs system [26] indicating that, the Rcs pathway elicits a global response to peptidoglycan stress [27].

Colicin M treatment also induced the expression of *cpxP*, which encodes the periplasmic inhibitor of the Cpx envelope stress response. The Cpx system appears to sense misfolded proteins that are synthesized for the periplasm, and it is controlled by the sensor kinase CpxA, the response regulator CpxR, and the periplasmic inhibitor CpxP. CpxP has been assumed to fine tune Cpx activation during envelope stress, by preventing its incorrect activation and enabling its rapid shut-down following envelope stress relief [28].

Treatment with colicin M also up-regulated a third cell envelope stress system, the *psp* genes that encode the membrane-bound phage shock proteins: PspA, PspB, PspC, PspD and PspG. The *psp* regulon consists of the *psp* operon with genes *pspA*, *pspB*, *pspC*, *pspD* and *pspE,* as well as genes *pspF* and *pspG*. Proteins PspB, PspC and PspD are located in the inner membrane, while PspA is on the cytoplasmic side of the inner membrane. In the absence of stress, PspA binds to protein PspF, thus inhibiting transcription of the *psp* operon. The *psp* genes are induced by filamentous phage infection and other stresses that impair cell membrane function [29,30]. This system is responsible for repair of inner membrane damage and maintenance of the proton motive force across the inner membrane [31,32]. Peptidoglycan damage provoked by colicin M exposes the sensitive inner membrane to osmotic damage requiring activation of membrane repair mechanisms.

### Colicin M induces expression of exopolysaccharide genes

Among the most strongly up-regulated genes, were those of the *wca* operon, which encodes the production of the exopolysaccharide, colanic acid [33]. The highly viscous colanic acid [34] is secreted into the extracellular environment to protect cells from osmotic stress such as provoked by cell envelope perturbations, including peptidoglycan damage or dessication [35]. In addition, colanic acid is involved in the later stages of biofilm formation; namely, the maturation and development of complex three-dimensional biofilm structures [24]. The *wca* operon is comprised of 19 genes that are involved in colanic acid synthesis from the nucleoside diphosphate sugars: GDP-L-fucose, UDP-d-glucose, UDP-d-galactose and UDP-D-glucuronate [36]. Colicin M treatment induced the expression of all 19 of the *wca* genes.

Exposure to colicin M also up-regulated the D-galactose transporter *galP*, as well as *galU*, which encodes the glucose-1-phosphate uridylyltransferase that is needed for UDP-glucose, an intermediate involved in the synthesis of colanic acid, trehalose, lipopolysaccharide and membrane-derived oligosaccharides [37].

Furthermore, our studies revealed strong induction of the *yjbEFGH* operon that is involved in the production of another, as-yet-unidentified, exopolysaccharide [38]. Recent studies have shown that the *yjbEFGH* operon is also induced by osmotic stress, and that the *wca* and *yjbEFGH* operons are negatively regulated by the general stress response sigma factor RpoS (σ^38^) [39]. Both the *wca* and the *yjbEFGH* operons are induced by the activated Rcs pathway to protect the bacterial cell from osmolysis.

### Colicin M induced additional osmotic and other stress responses

By inhibiting peptidoglycan synthesis, colicin M weakens membrane protection, provoking osmotic stress. Interestingly, genes c*reD*, *cbrA*, *cbrB* and *cbrC* of the CreB/CreC regulon were strongly induced already 30 min after exposure to colicin M. The Cre system was previously found to be involved in the switch from aerobic to anaerobic conditions. CreC is the sensor that also senses changes in the growth medium and/or metabolite pool levels, while CreB is a transcriptional regulator [40]. The two-component CreBC system positively controls transcription of *cbrA*. Recently, the CbrA protein was shown to protect against colicin M and osmotic shock, implying a function of CbrA in outer membrane structure [41]. Thus, it was proposed that in the natural environment with a limited supply of nutrients, CbrA protects sensitive cells from colicin M synthesized by competitors. Furthermore, CbrC, which we also found to be induced by colicin M treatment, has been shown to protect against colicin E2 and also seems to be involved in alteration of outer membrane structure [41]. Our results indicate that subinhibitory concentrations of colicin M could induce protection against colicins. Thus, in the natural environment, both colicin synthesis and the CreBC system are induced upon nutrient limitation [42,43]. Colicin produced in microbial communities by colicinogenic bacteria could in colicin sensitive community members induce protective responses. Moreover, activation of the CreBC two component regulator system was recently shown to play a major role in the ß-lactam resistance response [44] indicating that, subinhibitory concentrations of colicin M might elicit broader antimicrobial protection.

It can also be noted that more than 100 of the open reading frames up-regulated by colicin M treatment are classified as poorly characterized or with predicted functions. Among these, many are predicted membrane proteins and lipoproteins indicating that, to protect cells against peptidoglycan damage provoked by colicin M, an adaptive response to strengthen/stabilize the osmosensitive membrane is induced.

To resist the effects of colicin M treatment, other genes involved in the response to hyperosmotic stress were up-regulated; namely, *osmB* and *osmC*[45] as well as two inhibitors of C-lysozyme, *ivy* and a membrane bound and predicted lipoprotein *mliC*, were also induced by the Rcs system. Antibiotic-mediated peptidoglycan stress has also been shown to trigger expression of both of these genes [27].

Colicin M also induced other stress response genes, including *ydeI*, which is involved in hydrogen peroxide stress [46], as well as the *ibpA* and *ibpB* heat shock genes, which encode chaperones that can cooperate to prevent irreversible aggregation of proteins [47].

### Colicin M induces biofilm associated genes

In natural environments, bacteria often form biofilms, microbial communities in which bacteria adhere to an abiotic or biotic surface via surface charges as well as production of pili, fimbriae and exopolysaccharides. Microbial cells in biofilms show distinct properties, particularly resistance to antibiotics, disinfectants, shear stress and the immune system [48]. Biofilm formation proceeds in several tightly regulated steps: initial attachment, three-dimensional development by microcolony formation, biofilm maturation and the final step dispersal or cellular detachment to colonize other surfaces. Initially, flagella promote motility toward a surface; subsequently, flagella are lost and adhesive organelles such as curli fimbria enable attachment; and finally, colanic acid production promotes maturation into the three dimensional biofilm structure [49,50].

Colicin M treatment upregulated several genes involved in biofilm production. Thus, in addition to up-regulation of colanic acid, colicin M treatment up-regulated *rprA*, which encodes a small regulatory RNA affecting target mRNA translation. Expression of *rprA* has been shown to be activated by the Rsc system. RprA has been shown to repress *csgD*. This latter encodes the master transcriptional regulator that activates curli fimbriae and cellulose synthesis, both of which are involved in the initial stage of biofilm formation [51]. It has been postulated that by interfering with *csgD* mRNA translation, RprA might prevent the undesired co-expression of curli/cellulose and colanic acid [52]. In accordance, as we found upregulation of the colanic acid operon genes we also determined upregulation of the *omrA* and *omrB* genes, which encode two redundant small/antisense RNAs that have recently been shown to inhibit CsgD translation [53]*.*

Colicin M exposure up-regulated another biofilm-associated gene, *bdm*, which encodes the biofilm-dependent modulation protein. *Bdm* expression is positively regulated by RcsB in response to osmotic shock [25], and the Bdm protein has been recently shown to enhance biofilm formation [54].

The exposure to colicin M also upregulated *ydeH*, which codes for a diguanylate cyclase that can synthesize the second messenger bis-(3^′^-5^′^) cyclic di-guanosine monophosphate (c-di-GMP) [55-57]. *ydeH* is positively regulated by CpxR, and has been shown to inhibit motility as well as to promote adhesin and biofilm formation. In *E. coli*, c-di-GMP controls the synthesis of two exopolysaccharides: cellulose and poly-GlcNaC (PGA), a virulence factor of uropathogenic *E. coli*[58]. Our study thus showed that colicin M induced an envelope stress response which could provoke switching from a planktonic to a sessile lifestyle. Nevertheless, in crystal violet assays no induction of biofilm formation was observed (data not shown).

### Colicin M treatment downregulates flagellar biosynthesis genes

Not unexpectedly, among the down-regulated genes, there were in particular the genes involved in flagellar motility and in glutamine biosynthetic processes. In *E. coli*, flagellar expression and motility is controlled by the FlhDC complex that comprises >60 genes. Flagellar synthesis and function are processes that demand high energy consumption, and therefore, expression of the flagellar genes is tightly regulated [59]. In contrast to exopolysaccharide production, expression of the *flhDC* operon has been shown to be down-regulated by numerous environmental signals, such as high temperature, high osmolarity (concentrations of salts, in the presence of carbohydrates or low-molecular alcohols) and extreme pH [60,61].

Both the exopolysaccharide synthesis operons, *wca* and *yjbEFGH,* and the flagellar *flhDC* operon genes are controlled by the Rcs phosphorelay system. However, while the activated Rcs phosphorelay system induces exopolysaccharide synthesis, it down-regulates the *flhDC* operon due to repression by the RcsB cofactor RcsA.

### Validation of microarray results by real-time PCR

**Figure 3:**
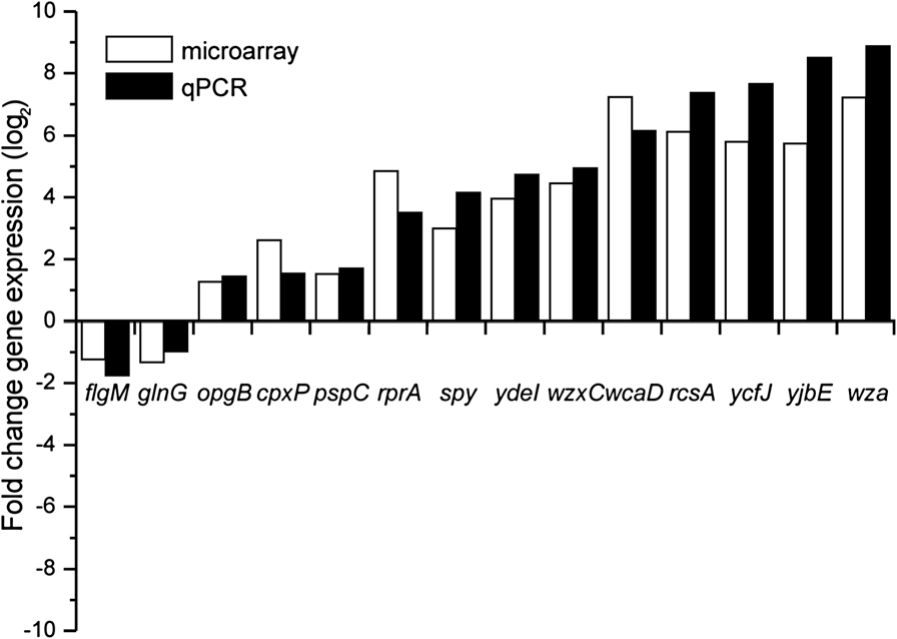
**Validation of the microarray results by qPCR.** Expression analysis of the 14 selected genes determined by microarray (open bars) and validated by qPCR (solid bars). The fold-changes for the microarray and qPCR were calculated as described (Materials and Methods) and represent gene expression levels following 60 min exposure to colicin M.

The gene expression results from the microarray analyses of total RNA isolated from cultures exposed to colicin M for 60 min were verified using qPCR. For this purpose, 14 genes differentially expressed upon colicin M treatment and from different functional groups, were selected: *ydeI, pspC, opgB, rprA, cpxP, ycfJ, rcsA, yjbE, wcaD, spy, wzxC, wza, glnG* and *wza*. For this comparison, the fold-changes of mRNA abundance of selected genes after 60 min colicin M exposure were plotted as those determined by qPCR *versus* those seen in the microarray analysis. The qPCR results confirmed differential gene expression observed by microarray analysis of the selected genes (Figure 3).

### Colicin M treatment does not promote significantly increased exopolysaccharide production

As the microarray data showed that the colanic acid operon genes were among the most strongly induced, a functional assay was performed to address whether the amount of colanic acid was changed accordingly. Production of colanic acid was quantified following exposure of *E. coli* to colicin M. Colanic acid was extracted from bacterial cultures treated with subinhibitory concentrations of colicin M for 60 min, 90 min and 120 min, as well as from an untreated control. While at the mRNA level there was significant induction of the *wca* operon genes, only a slight, 1.3-fold, increase in the production of colanic acid was seen at all sampling times. As an additional control, colanic acid was quantified from a culture overexpressing the *wca* operon encoded by a multicopy plasmid, pATC400 [62]. A 6-fold increase in colanic acid production was seen in comparison with an isogenic strain that did not overexpress the *wca* operon genes.

Treatment of *E. coli* with colicin M promotes the hydrolysis of the peptidoglycan lipid precursors, which results in the arrest of the polymerization steps and exposes the bacterial cells to envelope stress, which activates the Rcs and Cpx phosphorelay systems. Subsequently, cell motility is down-regulated, with induction of the expression of the exopolysaccharide *wca* and the *yjbEFGH* operon genes. Colicin M promoted hydrolysis of lipid II which prevents recycling of the lipid carrier for peptidoglycan synthesis and also limits its availability for exopolysaccharide biosynthesis, including colanic acid.

Following an initial growth stagnation (Figure 1 and also see Additional file 1: Figure S1), regrowth of cultures treated with these subinhibitory concentrations of colicin M indicate an adaptive response to the stress through the activation of the envelope and other stress responses. While neither an increase in colanic acid or biofilm production was detected, in the natural environment, activation of the various above-described stress responses might allow rapid adaptation, including mature biofilm formation, in the event of a sudden decrease/absence in antimicrobial concentrations.

### Genome-wide transcriptional analysis and other antimicrobials

A number of studies have shown that traditional antibiotics affect bacterial gene expression and physiology [1,2,63,64]. Thus, some β-lactam antibiotics that can also inhibit peptidoglycan synthesis have been shown to induce the production of colanic acid in *E. coli*, which indicates that these might exacerbate biofilm formation [65]. Investigation of the *E. coli* transcription profile in response to bactericidal concentrations of ampicillin also showed induction of the colanic acid biosynthetic pathway, as well as *rcsA*, the transcriptional activator of colanic acid synthesis and other stress responses [66]. However, the authors did not detect induction of the additional exopolysaccharide operon *yjbEFGH*, distinct from colanic acid. In *Staphylococcus aureus*, subinhibitory concentrations of β-lactams have been shown to up-regulate some virulence genes [67]*.* Moreover, the aminoglycoside tobramycin has been shown to induce biofilm formation in *E. coli* and in *Pseudomonas aeruginosa*, due to alterations in the levels of c-di-GMP [68]. Biofilm formation was also induced following exposure of *P. aeruginosa* to subinhibitory concentrations of tetracycline and norfloxacin [69].

Further to this, a number of studies have investigated the effects of antibiotics on the expression of the SOS regulon genes. Thus, the β-lactam antibiotic, ceftazidime, which is an inhibitor of a protein involved in cell wall biosynthesis, PBP3, has been shown to induce transcription of the *dinB* gene, which encodes the error-prone DNA polymerase Pol IV [70]. Subsequently, subinhibitory concentrations of ampicillin, norfloxacin and kanamycin were shown to induce mutagenesis due to antibiotic-mediated increases in reactive oxygen species, which results in SOS-induced mutagenesis that might lead to multidrug resistance [71]. An additional study showed that a number of antibiotics can promote an increase in mutation frequency; namely, ampicillin, ceftazidime, imipenem, ciprofloxacin, trimethoprim, sulfamethoxazole and tetracycline [72]. With the exception of imipenem, fosfomycin and tetracycline, the antibiotics tested were shown to induce *recA* expression, while inactivation of *recA* abolished the mutagenic effects.

In the present study, subinhibitory concentrations of colicin M did not induce the expression of *dinB* or *recA*. To further confirm that colicin M does not induce the SOS response, induction of the *sulA* gene following colicin M treatment was investigated. SOS-regulated SulA inhibits cell division by binding to FtsZ, which is required for septum formation. For this purpose, expression of the chromosomal *sulA-lacZ* fusion was studied in the ENZ1257 strain [73] without and with colicin M: no induction was detected (data not shown).

While colicins have been assumed to have roles in defense or invasion of an ecological niche, previous studies have shown that colicins are also involved in the promotion of microbial diversity within *E. coli* populations in the mammalian colon [9,74]. Furthermore, the nuclease colicins, E9 and E3, have been shown to have the potential to promote microbial genetic diversity via induction of the SOS response or via increased transcription of laterally acquired mobile elements, respectively [75].

Another study showed that colicins from one producer can induce production in another producer, thus resulting in colicin-mediated colicin induction [74]. Here, we show that subinhibitory concentrations of colicin M induced an envelope and other stress responses including the two component CreBC system connected with increased resistance to colicins M and E2. In natural environments, subinhibitory concentrations of colicin M could thus affect *E. coli* bacterial communities by promoting ecological adaptation enabling noncolicinogenic cells to survive and compete with colicin producers. The above-described phenomena might also be relevant in the natural settings of other bacterial species, as colicin M homologous proteins have been identified recently in human and plant pathogenic *Pseudomonas* species that have hydrolytic activity against peptidoglycan precursors [76]. Further, activation of the *P. aeruginosa* CreBC system has been shown to play a major role in the ß-lactam resistance response [44].

Resistance of pathogens to traditional antibiotics represents one of the greatest health care threats. The present lack of novel antibiotics is also of great concern. Colicin M has been recently shown to hydrolyse lipid II intermediates of Gram-negative and Gram-positive bacteria [12]. In addition, as the isolated colicin M catalytic domain displays full enzymatic activity, protein engineering can be used to allow binding and translocation in various Gram-negative and Gram-positive species [77,78]. Furthermore, low concentrations and low protein-to-bacteria ratios suffice for colicin M to kill *E. coli.* Targeting of lipid II has been indicated as a potential antibacterial strategy [79].

## Conclusion

In conclusion, subinhibitory concentrations of colicin M induced genes involved in adaptive responses to protect the population against envelope and other stresses, including the two component CreBC system associated with increased resistance to some colicins. Our study of the global transcriptional response to colicin M thus provides novel insight into the ecology of colicin M production in natural environments. While an adaptive response was provoked by colicin M treatment there was no induction of biofilm formation, SOS response genes, or other genes involved in mutagenesis, adverse effects shown to be promoted by a number of clinically significant traditional antibiotics. Thus, our study also corroborates the potential of colicin M as a promising antimicrobial and shows the value of microarrays for investigation of novel antimicrobials.

## Methods

### Colicin M expression and isolation

Prior to isolation of colicin M, the *cma* colicin M structural and *cmi* immunity genes were PCR amplified from the natural colicin M coding plasmid pCHAP1 using the primers ColM1 5^′^-TCACTCGAGCATGGAAACCTTAACTGTTCATGCA-3^′^ and ColM2 5^′^-CCACGCGTCCACTTCACAGTATGCTCACATTG-3^′^. The amplified fragment was digested with the *Xho*I and *Mlu*I restriction enzymes and cloned into the pET8c expression vector, also cut with the same two enzymes [80]. The isolated plasmid was designated pColM-imm Cloning of *cma* into the pET8c vector introduced an N-terminal histidine tag with expression under the control of a T7 promotor. Colicin M and the immunity protein were subsequently expressed in *E. coli* BL21 (DE3)pLysS and colicin M was purified using nickel affinity chromatography [80,81].

For large scale isolation an overnight culture of BL21 (DE3)pLysS, with plasmid pColM-imm was diluted 100 fold in 500 ml LB with ampicillin (120 μg/ml) and grown at 37°C to an OD_600_ 0.6-0.8, when chromosomal T7 polymerase production was induced by addition of 0.8 mM IPTG and incubated for a further 4 h. Subsequently, cells were harvested and resuspended in 50 mM phosphate, 300 mM NaCl buffer, pH 8, containing RNaseA (20 μg/ml), DNAse (10 μg/ml), lysozyme (1 mg/ml), 10 mM imidazole as well as protein inhibitors and incubated for 1 h at 4°C with shaking. The cells were then lysed with 3 min sonification, 40% amplitude and the supernatant obtained by centrifugation at 17000×g for 1 h at 4°C. The histidine-tag enabled Ni-NTA affinity column purification according to the user’s manual (Qiagen). Nonspecifically bound proteins were washed off the column with 50 mM phosphate, 300 mM NaCl, pH 8.0 buffer, containing 50 mM imidazole while colicin M was subsequently eluted with the same buffer containing 300 mM imidazole.

The colicin-M-containing fractions, as established by 10% SDS-PAGE, were then dialyzed against 5 mM phosphate buffer, pH 7.3, centrifuged at 17,000× *g* at 4°C for 30 min, and stored at −80°C. Colicin M purity was verified by SDS-PAGE (see Additional file 4: Figure S3), and (a concentration of 3.4 mg/ml) protein concentrations were determined using bicinchoninic acid protein assay kits (Pierce) and a Nanodrop ND 1000 spectrophotometer (Thermo Scientific). Finally colicin M was stored at −80°C.

### Growth conditions

The agar dilution method (National Committee for Clinical Laboratory Standards, 2000) was used to determine the minimal inhibitory concentration (MIC) of colicin M 50 ng/ml. For this purpose, an overnight culture of *E. coli* MG1655 [13] was diluted 1:625 in LB broth and grown at 37°C with aeration to an OD_600_ 0.6 when the culture was divided into several parts. One part served as a control while the other parts were treated with various concentrations of colicin M. The subinhibitory concentration (30 ng/ml) provoked temporary growth stagnation while growth decline was observed at higher concentrations, presumably due to cell lysis (see Additional file 1: Figure S1 in Additional file 2: Figure S2).

To investigate the effects of colicin M on the whole genome expression profile, an overnight culture of the *E. coli* strain MG1655 (F-lambda-*ilvG*-*rfb*-50 *rph*-1) was grown as described above. One part was treated with colicin M (30 ng/ml), while the untreated part served as the control. For gene expression analysis by microarray and qPCR, total RNA was isolated from 2-ml samples removed from each flask following 30 min and 60 min incubations at 37°C. The experiments were repeated at least two times.

For quantification of colanic acid, the growth conditions and the application of subinhibitory concentrations of colicin M were as described above. Colanic acid was purified from 50 ml cultures treated with colicin M for 60 min, 90 min and 120 min at 37°C, with aeration. The experiment was repeated at least two times.

### RNA isolation

Total RNA was extracted using the RNAProtect Bacteria Reagent (Qiagen) and RNeasy Mini kits (Qiagen), according to the manufacturer instructions. To remove residual DNA, on-column DNase digestion was performed during the RNA purification using the RNase-Free DNase Set (Qiagen). A Nanodrop ND 1000 spectrophotometer (Thermo Scientific) was used to confirm total RNA concentrations, while an Agilent 2100 Bioanalyser (Agilent Technologies, CA, USA) was used to evaluate the RNA quality. The isolated RNA was stored at −80°C until use.

### Microarray procedures

Gene expression analysis was performed using Affymetrix GeneChip® *E. coli* Genome 2.0 arrays. Target preparation, hybridization, washing, staining and scanning were performed as recommended by the Affymetrix GeneChip® Expression Analysis Technical Manual. The experiment was repeated at least two times.

The acquisition of array images and the data quality assessment were performed using an Affymetrix GeneChip Command Console. The GeneChip data was processed using several different R/Bioconductor packages. The Affymetrix raw data were normalized using the RMA algorithm from the XPS package. The data have been deposited in the NCBI Gene Expression Omnibus database (GEO, http://www.ncbi.nlm.nih.gov/geo) under GEO series accession number GSE37026.

Annotation of the genes and the data representation was managed using the ANNAFFY and AFFYCORETOOLS packages. The normalized data, converted to log_2_ values, were first limited to the ENTREZ-annotated probes from strain K12 (10208 probes). The remaining data were tested for differential expression, which was performed using the LIMMA package for the 30-min treated *versus* the 30-min untreated control and for the 60-min treated *versus* the 60-min untreated control bacterial culture. Differential expression was assessed using the 2-way factorial ANOVA model constructed using LIMMA package. Differential expression was assessed using the false discovery rate multiple test correction [82] and controlling type I error at α = 0.05. Differential expression of genes is represented as log_2_ ratios between the treated and untreated samples averaged over the two biological replicates. Important differentially expressed genes with log_2_ (fold change) greater than 1 or less than -1 denoting 2-fold up-regulated or down-regulated genes over time were considered for interpretation and are presented in Table 1. The expression of a subset of selected genes was validated by quantitative real-time PCR (qPCR) (see Additional file 5: Table S2).

### Real-time PCR

qPCR was performed for 14 genes that showed significant differential expression in the microarray analysis. Samples of 1 μg total RNA were reverse transcribed to synthesize cDNA using High Capacity cDNA Reverse Transcription kits (Applied Biosystems), according to the manufacturer instructions. qPCR was performed using the Power SYBR Green PCR Master Mix (Applied Biosystems) with an ABI PRISM 7900 HT Sequence Detection System (Applied Biosystems). The qPCR amplifications were performed as follows: 50°C for 2 min, 95°C for 10 min, followed by 40 cycles of 95°C for 15 s and 60°C for 1 min, and a final dissociation curve analysis step from 60°C to 95°C. Two negative reverse transcription controls were used to show no reverse transcription contamination. qPCR validation was performed on four biological replicates.

Publicly available sequences of the transcripts from the NetAffyx Analysis Centre (http://www.affymetrix.com/analysis/netaffx/index.affx) were analyzed to select target sequences, and the Primer3 software [83] and Primer Express 3.0 software (Applied Biosystems) were used for the design of the specific primers (Sigma). The primer sequences are listed in Additional file 5: Table S2.

Raw data were acquired using the Sequence Detection System software, version 2.3 (Applied Biosystems), and gene expression levels were analyzed using the 2^-δδ*CT*^ method [84], as the efficiency of the qPCR amplifications for all of the genes tested was >90%. geNorm [85] (available from medgen.ugent.be/~jvdesomp/genorm) was used to select the most stable genes, and out of the seven housekeeping genes tested, *lpp*, *aroE*, *gapA* were used as the reference genes, with their geometric mean used for normalization. The results are presented as log_2_ ratios between gene expression of treated and untreated cultures of four replicates, and they are presented as a comparison with the microarray data (Figure 3).

### Colanic acid quantification

Colanic acid was extracted from cultures grown and treated with colicin M as described above, and from untreated control cultures incubated under the same conditions. Colanic acid extraction and quantification was performed as described previously [86]. Briefly, for quantification, the amount of nondialyzable methylpentose *ω*-deoxyhexose (L-fucose), a component of colanic acid, was measured using a colorimetric reaction with authentic L-fucose (Sigma) as standard, and with concentrations ranging from 5 μg/ml to 100 μg/ml. Colanic acid levels were calculated as μg of nondialyzable *ω*-deoxyhexose/unit A600 nm/ml of culture.

### Biofilm production

The ability to form biofilms was investigated using crystal violet assays, as described previously [87]. To assess the induction of biofilm formation, 100 μl of overnight cultures were added to microtiter plates without and with colicin M, and incubated for 24 h at 37°C. The experiments were performed in triplicate.

### ß-Galactosidase assay

For quantification, the growth conditions and application of subinhibitory concentrations of colicin M are as described above. The ß-galactosidase activity of the *sulA-lacZ* gene fusion was measured as described previously [88].

## Competing interests

The authors declare that they have no competing interests.

## Authors’ contributions

Conceived and designed the experiments: DŽB. Performed the experiments: SK. Analyzed the data: SK, DŽB. Contributed reagents/materials/analysis tools: SK, DŽB. Wrote the paper: SK, DŽB. Both authors read and approved the final manuscript.

## Supplementary Material

Additional file 1: Figure S1 Growth of *E. coli* MG1655 treated with colicin M. The arrow denotes the time of addition of colicin M, at inhibitory (100 ng/ml, 50 ng/ml) and subinhibitory concentrations (30 ng/ml, 20 ng/ml, 10 ng/ml). Growth curves represent *E. coli* MG1655 cultures treated with different colicin M concentrations. Click here for file

Additional file 2: Figure S2 Effect of subinhibitory concentrations of colicin M on *E. coli* MG1566 viable counts. Growth curves with viable counts (CFU/ml as a function of time relative to antibiotic addition) are shown for untreated and treated culture (30 ng/ml of colicin M). Click here for file

Additional file 3: Table S1 Time course analysis of differentially expressed genes after 30 and 60 min exposure to subinhibitory concentrations of colicin M. p≤0.05, log2 FC≥1 and ≤−1, log2 FC≤1, ≥−1; *p≥0.05, log2 FC≥1 and ≤−1, log2 FC≤1, ≥−1.* Log2 FC values in bold correspond to log2 FC≥1 and ≤−1 when p≤0.05 and in regular type to log2 FC≤1, ≥−1 when p≤0.05. Log2 FC values in italics bold correspond to log2 FC≥1 and ≤−1 when p≥0.05 and in italics regular type to log2 FC≤1, ≥−1 when p≥0.05. Click here for file

Additional file 4: Figure S3 SDS-PAGE gel showing purity of isolated colicin M. Left, Protein ladder Page Ruler (Fermentas); Right, colicin M - 29.5 kDa, colicin M (3.4 mg/ml). Click here for file

Additional file 5: Table S2 Primer pairs used for qRT-PCR in the present study. Click here for file
