# Supplementary material for: Global transcriptional responses to the bacteriocin colicin M in Escherichia coli
Source: BMC Microbiol. 2013 Feb 19;13:42. doi: 10.1186/1471-2180-13-42 (PMC3599342; doi:10.1186/1471-2180-13-42)

**Figure S1** **Growth of *E. coli* MG1655 treated with various concentrations of colicin M.** The arrow denotes the time of addition of colicin M, at inhibitory (100 ng/ml, 50 ng/ml) and subinhibitory concentrations (30 ng/ml, 20 ng/ml, 10 ng/ml). Growth curves represent *E. coli* MG1655 cultures treated with different colicin M concentrations.


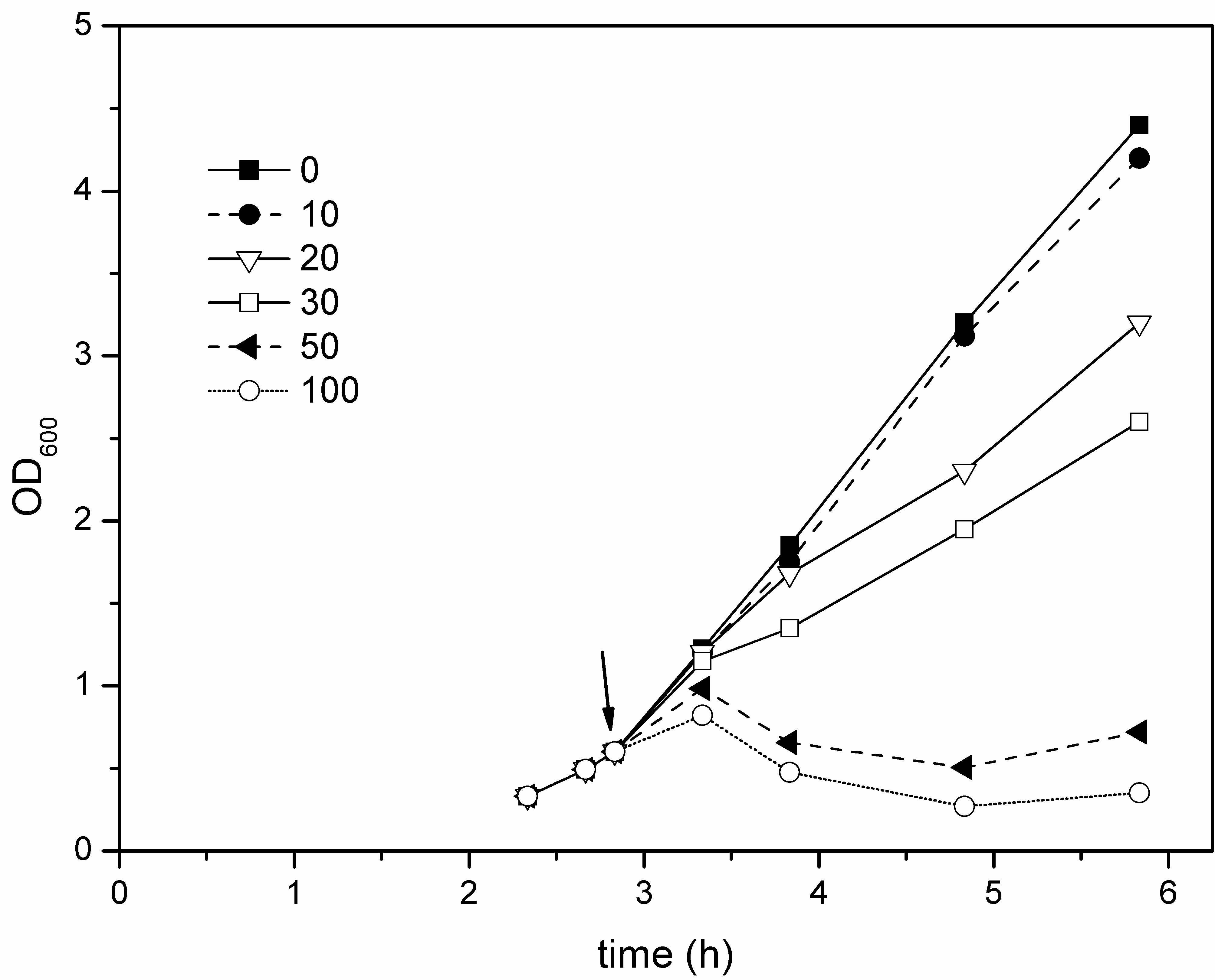

Supplement: Additional file 1: Figure S1 — Growth of E. coli MG1655 treated with colicin M. The arrow denotes the time of addition of colicin M, at inhibitory (100 ng/ml, 50 ng/ml) and subinhibitory concentrations (30 ng/ml, 20 ng/ml, 10 ng/ml). Growth curves represent E. coli MG1655 cultures treated with different colicin M concentrations. [file 1471-2180-13-42-S1.doc]
