# Supplementary material for: Global transcriptional responses to the bacteriocin colicin M in Escherichia coli
Source: BMC Microbiol. 2013 Feb 19;13:42. doi: 10.1186/1471-2180-13-42 (PMC3599342; doi:10.1186/1471-2180-13-42)

**Figure S2.** **Effect of subinhibitory concentrations of colicin M on *E. coli* MG1566 viable counts*.*** Growth curves with viable counts (CFU/ml as a function of time relative to antibiotic addition) are shown for untreated and treated culture (30 ng/ml of colicin M)**.**


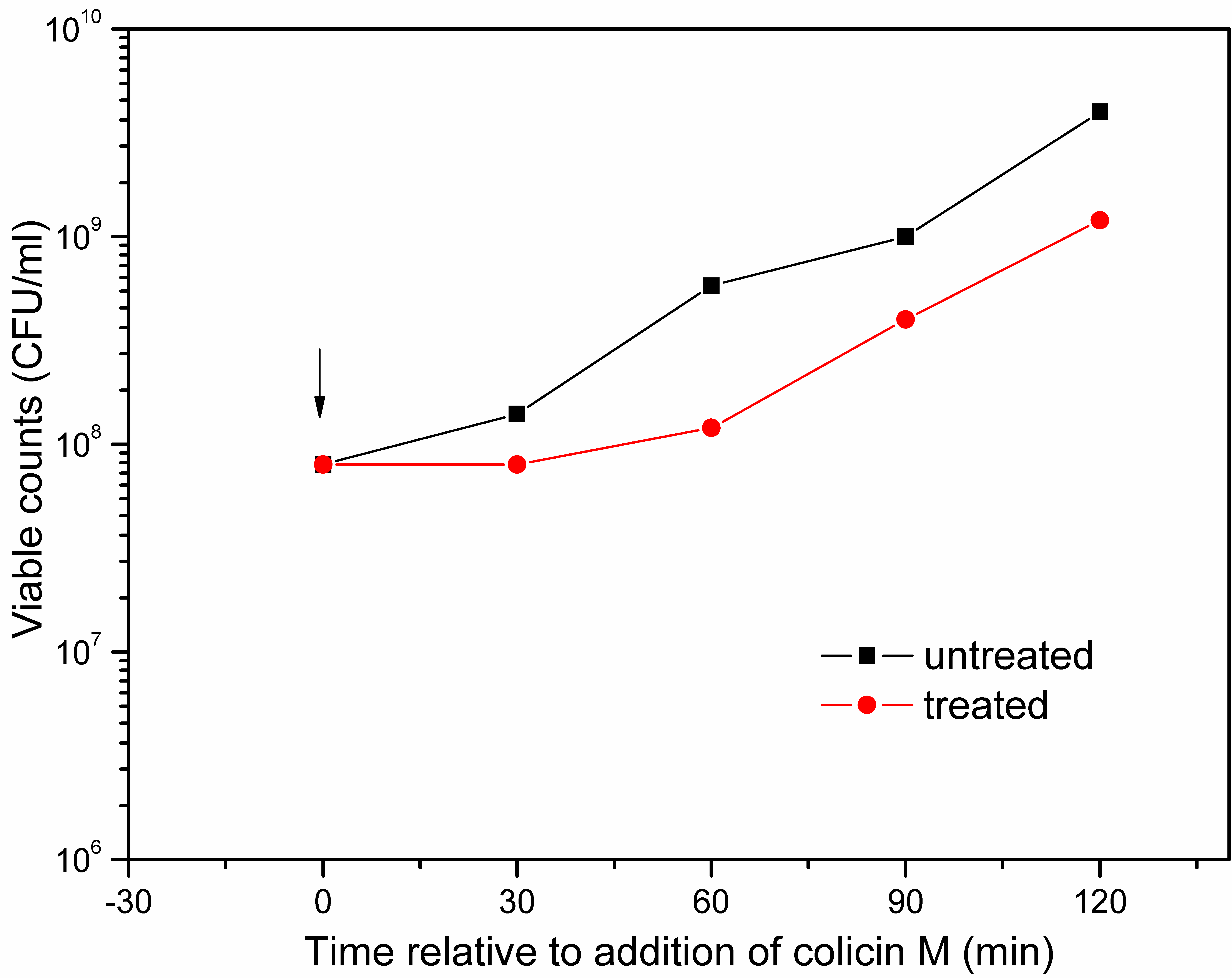

Supplement: Additional file 2: Figure S2 — Effect of subinhibitory concentrations of colicin M on E. coli MG1566 viable counts. Growth curves with viable counts (CFU/ml as a function of time relative to antibiotic addition) are shown for untreated and treated culture (30 ng/ml of colicin M). [file 1471-2180-13-42-S2.doc]
