# Supplementary material for: Global transcriptional responses to the bacteriocin colicin M in Escherichia coli
Source: BMC Microbiol. 2013 Feb 19;13:42. doi: 10.1186/1471-2180-13-42 (PMC3599342; doi:10.1186/1471-2180-13-42)
Supplement: Additional file 4: Figure S3 — SDS-PAGE gel showing purity of isolated colicin M. Left, Protein ladder Page Ruler (Fermentas); Right, colicin M - 29.5 kDa, colicin M (3.4 mg/ml). [file 1471-2180-13-42-S4.doc]

**Figure S3: SDS-PAGE gel showing purity of isolated colicin M.** Left, Protein ladder Page Ruler (Fermentas); Right, colicin M – 29,5 kDa, colicin M (3.4 mg/ml)

**
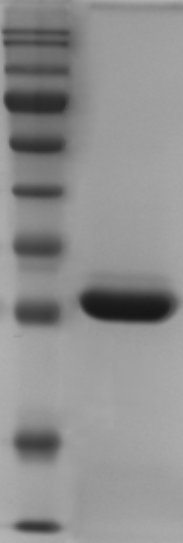
**

170 kDA .........

130 kDA .........

95 kDA ...........

72 kDA ............

56 kDA ............

43 kDA ............

34 kDA ............

26 kDA ............

17 kDA ...........

11 kDA ............
