# Supplementary material for: Global transcriptional responses to the bacteriocin colicin M in Escherichia coli
Source: BMC Microbiol. 2013 Feb 19;13:42. doi: 10.1186/1471-2180-13-42 (PMC3599342; doi:10.1186/1471-2180-13-42)
Supplement: Additional file 5: Table S2 — Primer pairs used for qRT-PCR in the present study. [file 1471-2180-13-42-S5.doc]

**Table S2** **Primer pairs used for qRT-PCR in the present study**

| **Target gene** | **Forward primer sequence (5’-3’)** | **Reverse primer sequence (5’-3’)** | **Amplicon size (bp)** | **Tm (°C)** |
| --- | --- | --- | --- | --- |
| *wzxC* | TCGGGCTAATGGTGGTGTC | CCATCAGCAGCGAACCAAT | 141 | 80,2 |
| *wza* | ttccgcctgcaaccttatg | cgctggctgtatccgtcat | 124 | 80,3 |
| *wcaD* | GGCATGGTGTGGAGTATTCGT | CCCGACCACCAGATAAAAGAAAC | 84 | 73,8 |
| *rcsA* | TgccgacgttatcattgagC | GATgaaacggtcttggctttg | 115 | 77,6 |
| *yjbE* | ttgccatatctgcgcttg | ggtggtcccggtattagaac | 191 | 87,0 |
| *ycfJ* | ACGCTCAGGTTGTTTCTGCAA | ACCCGGTAATGCGATTTTCA | 120 | 80,3 |
| *rprA* | CCCTGAGTGAAACAACGAATTG | ATCGTGGGAGATGGGCAAA | 53 | 73,4 |
| *spy* | CAGCGTGACCAGATGAAACG | CGGCGTCAGGATGTTGTAAA | 183 | 80,3 |
| *pspC* | tggtggtgctgtcgattttc | tcaccaaaggccatgttgtc | 106 | 78,6 |
| *flgM* | cgcctctgaagcctgtaagc | accgttacgaatcgccagtt | 200 | 84,4 |
| *glnG* | cggagagtacttcgcaaatgC | cggaaagcagattttgatga | 99 | 82,7 |
| *ydeI* | AggagaggaccgttacgttttTC | Ggctgaacttctcgtccatca | 90 | 77,1 |
| *cpxP* | TGAAGTCGGTTCAGGCGATAA | TCGAACATATGGCTCTGCGTACT | 75 | 78,4 |
| *opgB* | CAAGAGACGCTGGCAGTGAA | TGCTACGACCAAGTCCGAGAT | 97 | 80,1 |
| *gapA* | tcgtctggaaaaagctgcaa | tagcccagaacgcctttcat | 87 | 78,2 |
| *lpp* | Ctccagcaacgctaaaatcg | cgtcatctttagcagcctgaaC | 122 | 79,5 |
| *aroE* | GATCCCGTCATCGCTCATTC | TGCTCACACCATGCCAGAAA | 90 | 74,5 |
